# Platelet-derived CXCL12 regulates monocyte function, survival, differentiation into macrophages and foam cells through differential involvement of CXCR4–CXCR7

**DOI:** 10.1038/cddis.2015.233

**Published:** 2015-11-19

**Authors:** M Chatterjee, S N I von Ungern-Sternberg, P Seizer, F Schlegel, M Büttcher, N A Sindhu, S Müller, A Mack, M Gawaz

**Affiliations:** 1 Medizinische Klinik III, Kardiologie und Kreislauferkrankungen, Universität Tübingen, 72076 Tübingen, Germany; 2 Institute of Anatomy, Universität Tübingen, Neuroanatomie, 72074 Tübingen, Germany

## Abstract

Platelets store and release CXCL12 (SDF-1), which governs differentiation of hematopoietic progenitors into either endothelial or macrophage-foam cells. CXCL12 ligates CXCR4 and CXCR7 and regulates monocyte/macrophage functions. This study deciphers the relative contribution of CXCR4–CXCR7 in mediating the effects of platelet-derived CXCL12 on monocyte function, survival, and differentiation. CXCL12 and macrophage migration inhibitory factor (MIF) that ligate CXCR4–CXCR7 induced a dynamic bidirectional trafficking of the receptors, causing CXCR4 internalization and CXCR7 externalization during chemotaxis, thereby influencing relative receptor availability, unlike MCP-1. *In vivo* we found enhanced accumulation of platelets and platelet-macrophage co-aggregates in peritoneal fluid following induction of peritonitis in mice. The relative surface expression of CXCL12, CXCR4, and CXCR7 among infiltrated monocytes was also enhanced as compared with peripheral blood. Platelet-derived CXCL12 from collagen-adherent platelets and recombinant CXCL12 induced monocyte chemotaxis specifically through CXCR4 engagement. Adhesion of monocytes to immobilized CXCL12 and CXCL12-enriched activated platelet surface under static and dynamic arterial flow conditions were mediated primarily through CXCR7 and were counter-regulated by neutralizing platelet-derived CXCL12. Monocytes and culture-derived-M1–M2 macrophages phagocytosed platelets, with the phagocytic potential of culture-derived-M1 macrophages higher than M2 involving CXCR4–CXCR7 participation. CXCR7 was the primary receptor in promoting monocyte survival as exerted by platelet-derived CXCL12 against BH3-mimetic induced apoptosis (phosphatidylserine exposure, caspase-3 activation, loss of mitochondrial transmembrane potential). In co-culture experiments with platelets, monocytes predominantly differentiated into CD163^+^ macrophages, which was attenuated upon CXCL12 neutralization and CXCR4/CXCR7 blocking antibodies. Moreover, OxLDL uptake by platelets induced platelet apoptosis, like other platelet agonists TRAP and collagen-related peptide (CRP). CXCL12 facilitated phagocytosis of apoptotic platelets by monocytes and M1–M2 macrophages, also promoted their differentiation into foam cells via CXCR4 and CXCR7. Thus, platelet-derived CXCL12 could regulate monocyte-macrophage functions through differential engagement of CXCR4 and CXCR7, indicating an important role in inflammation at site of platelet accumulation.

Platelets are central players in regulation of inflammation at the site of thrombosis.^1, 2, 3^ When platelets are activated they release a variety of pro-inflammatory mediators including the chemokine CXCL12 (SDF-1).^4, 5, 6, 7^ CXCL12 binds to its chemokine receptors CXCR4 and CXCR7 and regulates cell migration, adhesion and survival.^8, 9, 10, 11^

Recently, platelets have been recognized to store substantial amounts of CXCL12 in their alpha-granules and release the chemokine upon activation.^5, 6^ Platelet-derived CXCL12 propagates migration and subsequent differentiation of CD34^+^ progenitor cells^5, 12^ into either an endothelial or a macrophage/foam cell phenotype depending on the culture conditions.^12, 13^ Release of CXCL12 from platelets is enhanced in acute coronary syndromes and correlates with the number of circulating CD34^+^ progenitor cells and platelet/CD34^+^ co-aggregates.^14, 15^ Enhanced levels of platelet–CXCL12 are associated with preservation of left ventricular function following myocardial infarction in humans.^16^ Administration of recombinant CXCL12 preserves myocardial function following transient ischemia in mice.^17^

Monocytes/macrophages have a critical role in vascular inflammation and disease progression of atherosclerosis.^18^ Monocytes express both CXCR4 and CXCR7 although their role in monocyte function is still incompletely understood.^9, 19^

In the present study, we explored the effect of platelet-derived CXCL12 on monocyte function and the differential role of CXCR4 and CXCR7 for monocyte function and differentiation. We found that both chemokine receptors have a decisive but differential role for platelet-dependent monocyte function.

## Results

### Surface expression of CXCR4 and CXCR7 is dynamically regulated on migrating monocytes

**Figure 1:**
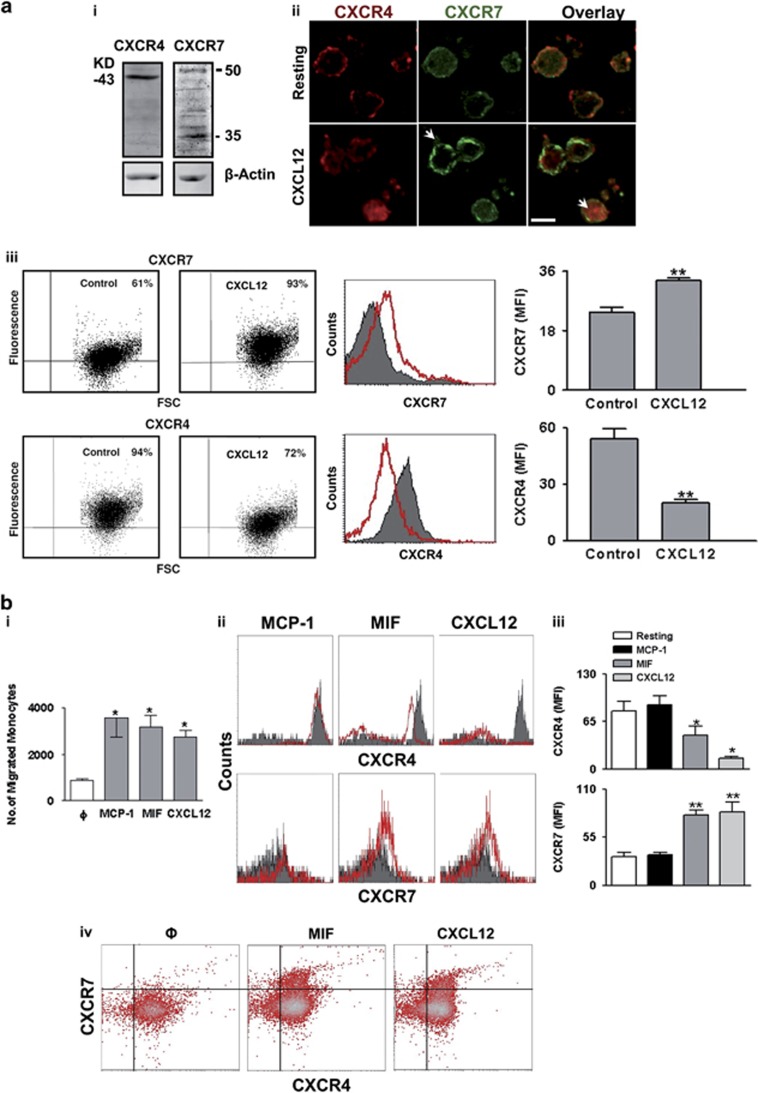

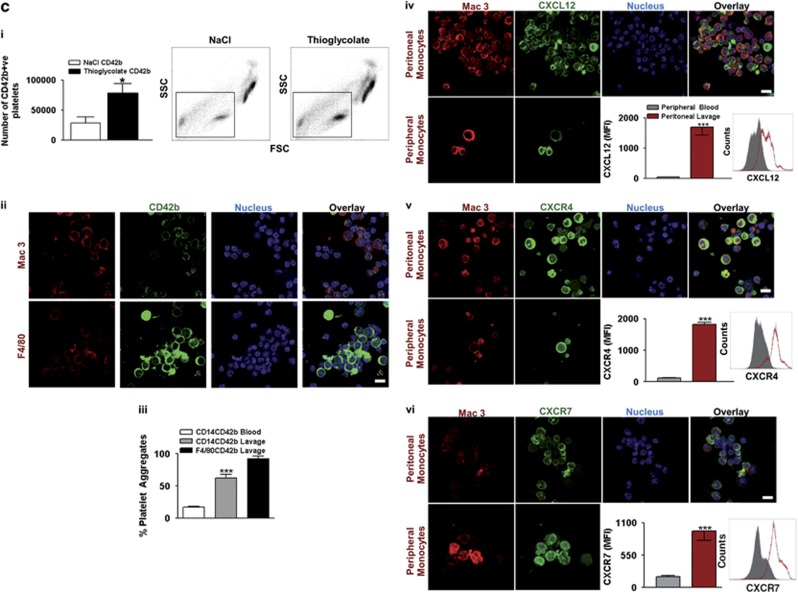
CXCL12 induces internalization of CXCR4 and surface translocation of CXCR7 conferring differential receptor availability on monocyte surface. (**a**) (**i**) Western blot analysis for the expression of CXCR4 (immunoreactive band at 43 KD) and CXCR7 (at 35, 50 KD) in untreated resting monocytes. Data are representative of three independent western blot analysis performed with monocytes isolated from three independent healthy donors. (**ii**) Representative immunofluorescence confocal microscopic images showing relative intracellular localization of CXCR4 (Alexa Fluor 647-red) and CXCR7 (Alexa Fluor 488-green) in untreated and CXCL12-treated (1 *μ*g/ml) monocytes. Internalized CXCR4 and externalized CXCR7 among CXCL12-treated monocytes are indicated by white arrows. Bar=5 *μ*m. Images are representative of three independent experiments performed with three healthy donors. Relevant control stainings are shown in Supplementary Figure 1. (**iii**) Flow-cytometric dot plot (left) showing percentage of CXCR4^+^ and CXCR7^+^ monocytes under resting conditions (94 and 61%, respectively) and following CXCL12 (1 *μ*g/ml) treatment, causing downregulation of CXCR4^+^ monocytes owing to receptor internalization and an increase in CXCR7^+^ monocytes due to enhanced surface exposure. Flow-cytometry histograms showing the relative surface expression of CXCR4 and CXCR7 in the presence (red) and absence (gray) of CXCL12. Corresponding bar diagram shows a significant (**P<0.01) increase in MFI for CXCR7-PE in CXCL12-treated monocytes and a concomitant (**P<0.01) decrease in MFI for CXCR4-PE. Data represent mean±S.E.M. of five independent experiments. (**b**) (**i**) Bar diagram showing the relative number of monocytes migrated in response to CXCL12 (1 *μ*g/ml), MIF (200 ng/ml), and MCP-1 (50 ng/ml). *P<0.05 as compared with control. Data are mean±S.E.M. of four independent chemotaxis analyses with monocytes from three donors. (**ii**) Flow-cytometric histograms showing the relative surface expression of CXCR4 and CXCR7 on the surface of migrated monocytes in response to CXCL12, MIF and MCP-1. Gray fills indicate control or basal level of expression and red lines indicate monocytes migrated toward CXCL12, MIF and MCP-1. (**iii**) Bar diagram showing surface expression of CXCR4 and enhanced surface expression of CXCR7 among monocytes following migration in response to CXCL12 and MIF but not MCP-1. Data represent mean±S.E.M. of three independent chemotaxis analyses with monocytes from four donors. (**iv**) Flow-cytometric density plot showing the relative surface expression of CXCR4 and CXCR7 on migrating monocytes showing relative surface expression of CXCR4–CXCR7 in response to CXCL12 or MIF as compared with control. (**c**) (**i**) Bar diagram of flow-cytometric data showing the number of platelets as detected in the peritoneal fluid collected from mice following induction of peritonitis by thioglycolate as compared with control injection of NaCl. Data represent mean±S.E.M. derived from seven mice in each group. *P<0.05 as compared with NaCl-treated group. **Right,** Flow-cytometric dot plot of FSC-SSC showing infiltrated platelets in the peritoneal lavage as indicated within the square and in conjugation or aggregate formed with other cells. (**ii**) Immunofluorescence confocal microscopic images showing the presence of Mac-3^+^ monocytes (in red) and F4/80 labelled macrophages (in red) in combination with CD42b^+^ platelets (in green). Bar=5 *μ*m. (**iii**) Bar diagram of flow-cytometric data showing the relative percentage of monocyte platelets (CD14^+^CD42b^+^) and macrophage platelet (F4/80^+^CD42b^+^) aggregates detected in peripheral blood and peritoneal lavage following thioglycollate-induced peritonitis in mice. ***P<0.001 as compared with monocyte platelet aggregates detected in peripheral blood. Data represent mean±S.E.M. derived from seven mice in each group. Immunofluorescence confocal microscopic images showing the expression of CXCL12 (**iv** in green), CXCR4 (**v** in green), and CXCR7 (**vi** in green) among Mac-3+ (in red) peripheral blood monocytes and infiltrated peritoneal monocytes following induction of peritonitis in mice. Bar=5 *μ*m

Surface expression of CXCR7 on monocytes is a widely debated issue.^9, 19^ Monocytes express both CXCR4 and CXCR7 (Figures 1ai–iii). The CXCR4/7 ligand CXCL12 induced a reduction in surface expression of CXCR4 due to receptor internalization (Figures 1aii and iii) thereby reduced the percentage of CXCR4^+^ monocytes. In contrast, there was a concomitant enhanced surface expression of CXCR7 resulting in increased percentage of CXCR7^+^ monocytes (Figure 1a). Thus, surface presentation of CXCR4–CXCR7 on monocytes is dynamically regulated and dependent on the presence of CXCL12, as previously found for platelets.^8^

Next, the dynamic surface expression of CXCR4–CXCR7 on migrating monocytes was analyzed. As comparison, MCP-1 (CCL2) that promotes monocyte migration but does not interact with CXCR4 or CXCR7 was evaluated. Both CXCR4/7 ligands, CXCL12 and MIF, induced monocyte migration comparable to MCP-1 (Figure 1bi). Migration toward CXCL12 and MIF caused CXCR4 internalization and enhanced CXCR7 surface expression (Figures 1bii and iii) whereas MCP-1 did not modulate CXCR4–CXCR7 surface expression on migrating monocytes. Therefore, the initial chemotactic response toward CXCL12 and MIF alters the surface availability of CXCR4–CXCR7, which then regulates the relative involvement of CXCR4 and CXCR7 in subsequent functional events.

### Surface expression of CXCL12, CXCR4, and CXCR7 is altered on infiltrating monocytes during peritonitis

To decipher the significance of platelet–monocyte associations under inflammatory or infectious conditions, thioglycollate-induced peritonitis model in mice was utilized. CD42b^+^ platelets could be detected among the infiltrated cells of peritoneal lavage (Figure 1ci), also in association with CD14^+^ monocytes and F4/80^+^ macrophages (Figures 1cii and iii). The relative number of infiltrating CD42b^+^ platelets (Figure 1ci) and the surface expression of CXCL12 (data not shown) were more in thioglycollate-induced peritoneal lavage as compared with control; further, the percentage of platelet-monocyte/macrophage aggregates was more among peritoneal cells as compared with peripheral blood. The relative surface expression of CXCL12, CXCR4, and CXCR7 was also enhanced among infiltrated monocytes as compared with peripheral blood (Figures 1aiv–vi and Supplementary Figure 2 for macrophages).

### CXCR4 but not CXCR7 mediates platelet–CXCL12-dependent monocyte migration

**Figure 2:**
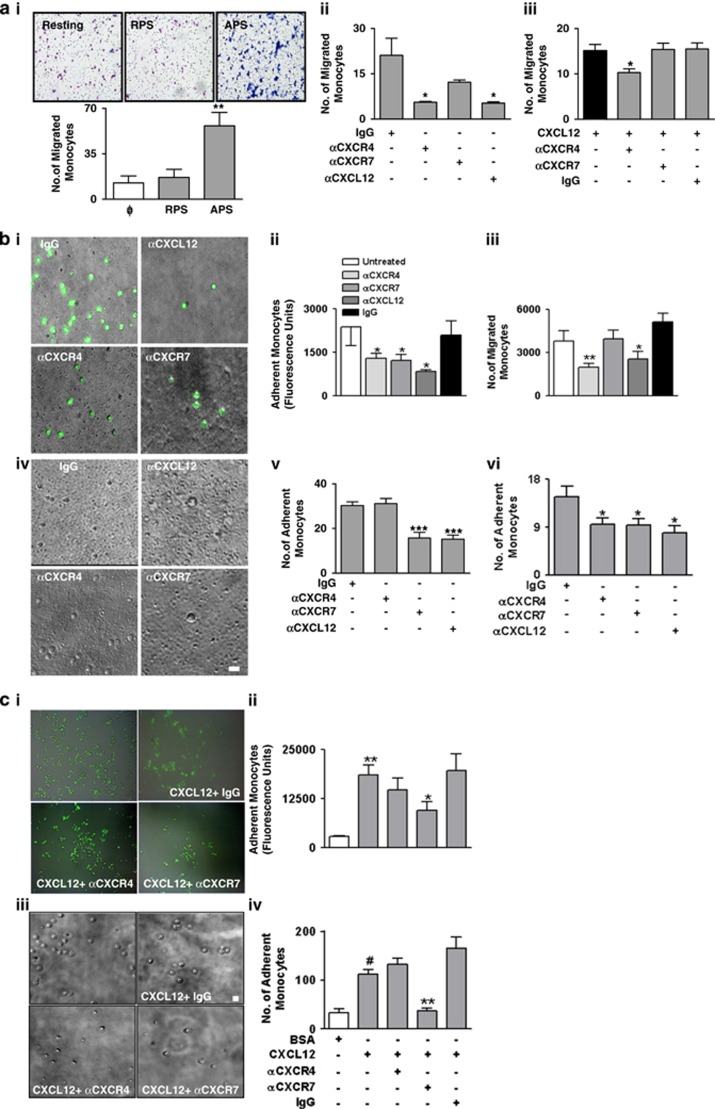
Platelet-derived CXCL12 regulates monocyte functions (chemotaxis, adhesion) through differential involvement of CXCR4–CXCR7. (**a**) (**i**) Images of the trans-membrane of the boyden chamber chemotaxis assay stained with May-Grünwald/Giemsa showing the migrated monocytes in response to resting and activated platelet supernatant (RPS and APS). Images were captured from randomly selected microscopic fields at × 20 magnification. Bar diagram showing a significantly enhanced (**P<0.01) chemotactic migration of peripheral blood monocytes toward APS as compared with RPS. (**ii**) APS-driven chemotaxis of monocytes was substantially (*P<0.05 as compared with IgG-treated APS) decreased following treatment of APS with a neutralizing antibody against CXCL12 (10 *μ*g/ml) before initiation of chemotaxis and following blocking of CXCR4 (10 *μ*g/ml) (*P<0.05 as compared with IgG) on monocytes before initiation of chemotaxis. (**iii**) Bar diagram showing rCXCL12-initiated chemotaxis of monocytes was substantially (*P<0.05 as compared with IgG) decreased following blocking of CXCR4 (10 *μ*g/ml) on monocytes before initiation of chemotaxis. Data represent mean±S.E.M. of five independent chemotaxis analysis with monocytes from five healthy donors. (**b**) Original immunofluorescence (**i**) and (**iv**) brightfield images showing the static adhesion of monocytes to activated platelets adherent on collagen coated (100 *μ*g/ml) surface (resembling platelets adhered to exposed sub-endothelial matrix components) in the presence/absence of a neutralizing antibody (10 *μ*g/ml) against platelet-derived CXCL12, and blocking antibody against CXCR4 and CXCR7 (10 *μ*g/ml) on monocyte surface with respect to IgG control. Bar=5 *μ*m. (**ii**) Bar diagram representing the number of monocytes migrated toward collagen adherent activated platelet-derived CXCL12 in the lower chamber of a transwell migration chamber and (**iii**) following their adhesion to activated platelets in the lower chamber. Neutralizing CXCL12 from activated platelet surface or blocking CXCR4–CXCR7 on monocytes significantly reduced (**P<0.001 and **P<0.001 as compared with IgG) the number of monocytes adhering to activated platelets. Data represent mean±S.E.M. of three independent experiments. (**v**) Bar diagram representing the number of monocytes following their adhesion to activated platelets under static conditions. Neutralizing CXCL12 from activated platelet surface or blocking CXCR7 on monocytes significantly reduced (***P<0.001 as compared with IgG) the number of monocytes adhering to activated platelets. Data represent mean±S.E.M. of three independent experiments. (**vi**) Bar diagram denoting the number of monocytes adhering to immobilized platelets on collagen-coated surface at arterial sear rates. Neutralizing CXCL12 from activated platelet surface or blocking CXCR7 on monocytes significantly reduced (*P<0.05 respectively as compared with IgG) the number of monocytes adhering to activated platelets. Data represent mean±S.E.M. derived from five independent flow chamber experiments. (**c**) Original immunofluorescence (**i**) and (**iii**) brightfield images (Bar=5 *μ*m) showing the static adhesion of monocytes to rCXCL12-coated (200 ng/ml) surface in the presence/absence of blocking antibody against CXCR4 and CXCR7 (10 *μ*g/ml) on monocyte surface with respect to IgG control. (**ii**) Bar diagram showing a significantly enhanced (**P<0.01) response for fluorescent monocytes adhered to a CXCL12- (200 ng/ml) coated surface as compared with control (5% BSA coated). Static adhesion of monocytes to immobilized CXCL12 was significantly reduced (*P<0.05 as compared with IgG) following blocking of CXCR7 (10 *μ*g/ml) on monocytes before adhesion. Data represent mean±S.E.M. of three independent experiments with monocytes from three donors. (**iv**) Bar diagram of following manual counting of adherent monocytes showing a significantly enhanced (^#^*P*<0.05) number of monocytes adhered to a CXCL12-(200 ng/ml) coated surface as compared with control (5% BSA coated). Static adhesion of monocytes to immobilized CXCL12 was significantly reduced (**P<0.01 as compared with IgG) following blocking of CXCR7 (10 *μ*g/ml) on monocytes before adhesion. Data represent mean±S.E.M. of three independent experiments with monocytes from three donors

Platelets accumulate and adhere to sites of vascular or tissue injury, release CXCL12, and thereby stimulate recruitment of inflammatory cells.^4, 5, 7^ Currently, supernatant of platelets adhering to collagen was generated and monocyte migration was analyzed. Supernatant form activated (APS) but not resting platelets (RPS) significantly enhanced monocyte migration (Figure 2ai). APS-induced monocyte migration was dependent on CXCL12, since neutralizing anti-CXCL12 mAb significantly inhibited (*P*<0.05) monocyte migration toward APS (Figure 2a). Further, following blocking of CXCR4 but not CXCR7, monocyte migration toward APS was significantly reduced (*P*<0.01) (Figure 2aii). Similarly, the chemotactic response of monocytes toward recombinant CXCL12 was mediated through CXCR4 and therefore counteracted by blocking antibody against CXCR4 but not CXCR7 (*P*=0.2079) (Figure 2aiii). Thus, platelet-derived CXCL12 is a potent chemotactic factor for peripheral blood primary monocytes and elicits its migratory response primarily through CXCR4 and not CXCR7.

### CXCR7 primarily mediates monocyte adhesion to CXCL12-enriched platelet surface

Platelets surface expresses substantial amounts of CXCL12 and induces chemotactic recruitment of progenitor cells.^4, 5^ Currently, monocyte migration toward collagen adherent platelets and their subsequent adhesion to activated platelet surface was ascertained. Neutralizing CXCL12 from activated platelet surface blocking either CXCR4 or CXCR7 on monocytes significantly inhibited both migration (Figures 2bi–iii) and subsequent adhesion of monocytes to activated platelets. In a static adhesion assay, isolated monocytes were directly added to collagen-adherent platelets in the presence of anti-CXCL12, anti-CXCR4/-7 mAb, or IgG control. We found that neutralizing CXCL12 from adherent platelets and prior-blocking of CXCR7 on monocytes with anti-CXCR7 but not using anti-CXCR4 inhibited monocyte adhesion to immobilized platelets (Figures 2biv–v). Similar results were observed for monocyte adhesion onto collagen adherent immobilized (CXCL12 enriched) platelets under dynamic arterial flow rate conditions. In the presence of a neutralizing antibody against platelet-derived CXCL12 and blocking antibody against CXCR7 but not CXCR4 or IgG control monocyte adhesion to immobilized platelets was significantly reduced (*P*<0.05) (Figure 2bvi), indicating that CXCR7 primarily mediates monocyte adhesion to immobilized platelets enriched in CXCL12. Moreover, monocyte adhesion to immobilized recombinant CXCL12 was dependent on CXCR7 but not on CXCR4 as shown by antibody-based adhesion experiments (Figures 2ci–iv).

### CXCL12 promotes phagocytosis of platelets by monocytes and macrophages through both CXCR4 and CXCR7

**Figure 3:**
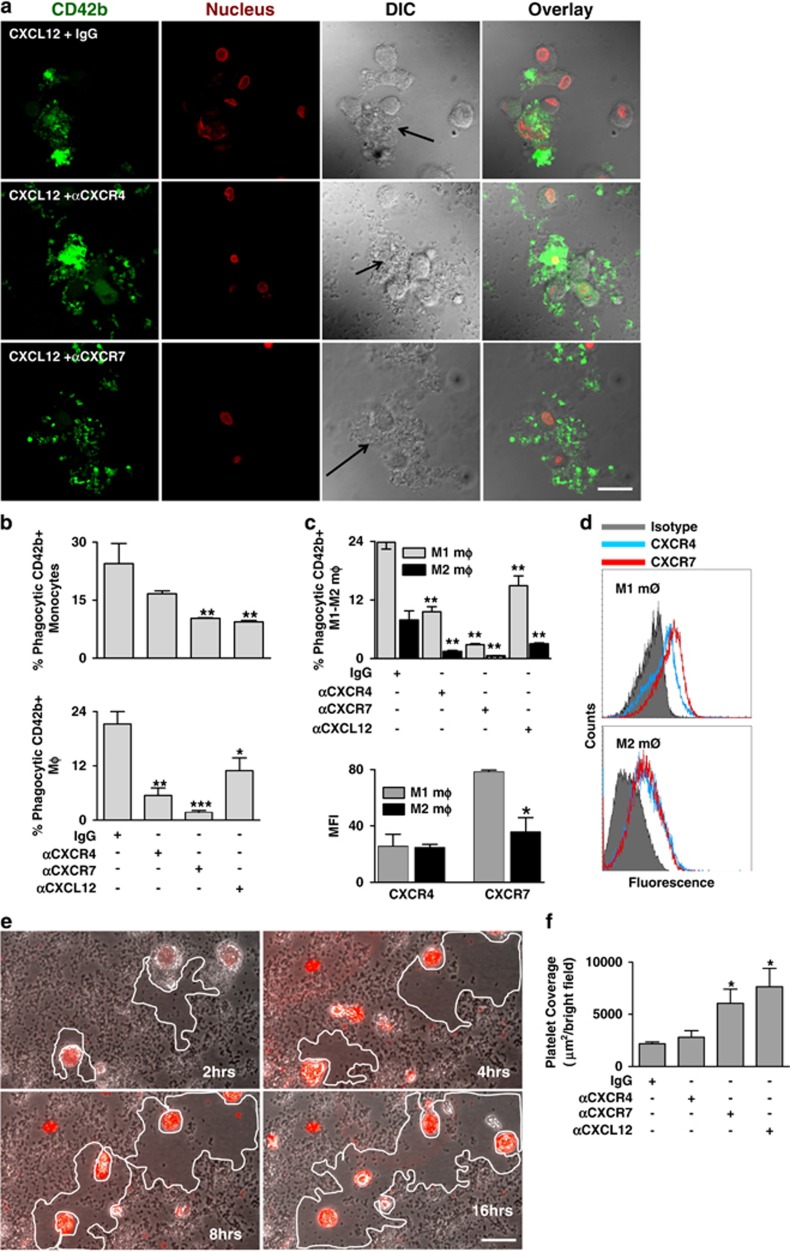
Platelet-derived-CXCL12 regulates monocyte phagocytosis, thrombus resolution through differential involvement of CXCR4–CXCR7. (**a**) Representative immunofluorescence confocal microscopic images showing a qualitative estimate of the phagocytic uptake of CD42b-FITC (platelet specific marker in green) labelled platelets by peripheral blood monocytes treated with rCXCL12 (1 *μ*g/ml), and following blocking CXCR4 or CXCR7 (10 *μ*g/ml) on monocyte surface with respect to IgG. Monocytes showing shape change following phagocytic uptake of platelets are shown with arrows in the upper and middle panels, whereas monocytes surrounded by platelets in vicinity but not exhibiting phagocytic uptake are indicated in the lower panel. Bar=5 *μ*m. (**b**) Bar diagram representing the quantitative estimate of phagocytic uptake of CD42b^+^ platelets by peripheral blood monocytes and culture-derived macrophages treated with rCXCL12 (1 *μ*g/ml), which was significantly reduced in presence of a CXCL12 neutralizing antibody (10 *μ*g/ml) (*P<0.05 and **P<0.05 as compared with IgG) and following blocking of CXCR4 and particularly CXCR7 (10 *μ*g/ml) (**P<0.01 and ***P<0.001 as compared with IgG) on monocyte and macrophages before initiation of phagocytosis. (**c**) Bar diagram showing phagocytic uptake of platelets by culture-derived M1–M2 macrophages treated with rCXCL12 (1 *μ*g/ml), which was significantly reduced in the presence of a CXCL12 neutralization (**P<0.01 as compared with IgG) and following blocking of CXCR4 and particularly CXCR7 (**P<0.01 as compared with IgG). (**d**) Flow-cytometric histogram overlay showing the relative surface expression of CXCR4 and CXCR7 on M1 and M2 macrophages. **Lower left**, bar diagram of flow-cytometric data showing the relative surface expression of CXCR4 and CXCR7 on culture-derived M1 and M2 macrophages. *P<0.05 for CXCR7 surface expression between M1 and M2 macrophages. Data are derived from four independent experiments performed with culture-derived M1–M2 macrophages. (**e**) Representative fluorescence images showing the phagocytic clearance or resolution of platelet covered surface by monocytes in culture (2, 4, 8, and 16 h). Bar=10 *μ*m. (**f**) Bar diagram representing resolution of thrombus (reduction in thrombus area or platelet coverage) following phagocytic clearance by monocytes. Platelet covered area was significantly reduced in the presence of monocytes, which was counteracted in the presence of CXCR7 (10 *μ*g/ml) blocking antibody. Neutralizing CXCL12 (10 *μ*g/ml) also counteracted phagocytic clearance of thrombus (*P<0.05 as compared with IgG). Data represent mean±S.E.M. of three independent experiments

CXCL12 and CXCR7 agonists trigger bacterial phagocytosis by monocytes.^9^ Currently, we found that CXCL12 stimulates phagocytosis of CD42b^+^ platelets by both monocytes and macrophages, which were significantly inhibited in the presence of blocking anti-CXCR4 and anti-CXCR7 mAb (Figures 3a and b). Phagocytic activity was dependent on the subtype of macrophages (M1, M2). Phagocytosis of platelets by the culture-derived pro-inflammatory M1 macrophage phenotype (CD86^+^) was higher than that of the M2 (CD163^+^) (Figure 3c) phenotype. Phagocytic activity of M1 and M2 macrophages was associated with their relative levels of CXCR7 surface expression. M1 macrophages showed significantly enhanced CXCR7 surface expression as compared with M2, whereas surface expression of CXCR4 did not differ between M1 and M2 (Figure 3d). To further evaluate the phagocytic activity, monocytes were co-cultured with isolated platelets (1 : 200) and phagocytosis of platelets was evaluated at specific time intervals (2, 4, 8, 16 h) as shown in Figure 3e. In another set of experiments, monocytes were incubated with thrombus formed on collagen-coated surface for overnight at 37 °C. Monocytes effectively contributed to platelet clearance as deciphered by a reduction in the area covered by platelets over time (Figures 3e and f). In the presence of anti-CXCR7 and anti-CXCL12 but not anti-CXCR4 or IgG control platelet thrombus clearance by monocytes was significantly reduced (Figure 3f).

### Platelet-derived CXCL12 promotes survival of monocytes through CXCR7

**Figure 4:**
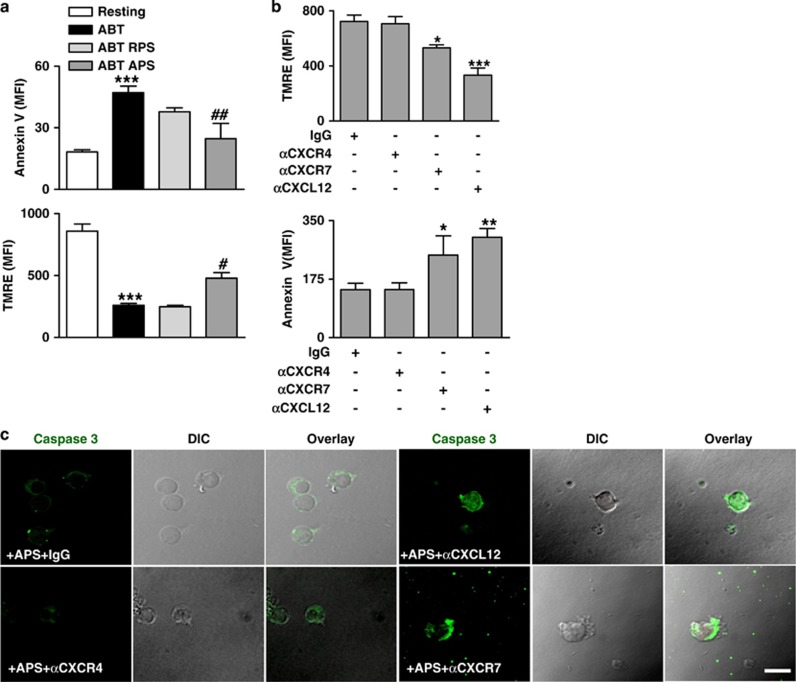
Platelet-derived-CXCL12 promotes survival of monocytes through CXCR7. (**a**) Bar diagram showing MFI of TMRE fluorescence denoting mitochondrial transmembrane potential loss (ΔΨm) and Annexin V binding denoting externalization of phosphatidylserine among resting monocytes, following significant (***P<0.001 as compared with resting) induction of apoptosis by BH3-mimetic ABT-737 (25 *μ*M). APS significantly (^##^*P*<0.01, ^#^*P*<0.05 as compared with ABT-737 treated) decreased ABT-737-induced apoptosis in monocytes. (**b**) Bar diagram showing MFI of TMRE fluorescence and Annexin V binding among apoptotic monocytes. The anti-apoptotic effect of APS on ABT-737-induced monocyte apoptosis was substantially abrogated (*P<0.05 compared with IgG) in the presence of anti-CXCR7, but not anti-CXCR4 antibody (10 *μ*g/ml). Neutralizing CXCL12 (10 *μ*g/ml) in APS also reduced (***P<0.001, **P<0.01 as compared with IgG) the anti-apoptotic effect of APS against ABT-737-induced apoptosis. Data are mean±S.E.M. from three independent experiments. (**c**) Representative immunofluorescence images showing relative presence/absence of active-cleaved caspase 3 (in Alexa Fluor-488-green) in resting and apoptotic monocytes following ABT-737 treatment and in the presence of APS in combination with/without blocking antibodies against CXCR4–CXCR7 and neutralizing antibody against platelet-derived CXCL12 as indicated (Bar=5 *μ*m). Images are representative of three independent experiments

Platelet interaction with monocytes prolongs their survival.^20^ CXCL12 is a survival factor for a variety of CXCR7^+^ cells.^21, 22, 23^ We found that supernatant of activated (APS) but not resting (RPS) platelets significantly attenuated ABT (BH3-mimetic)-induced apoptosis in monocytes (Figure 4a) as indicated by reduced expression of activated caspase-3, surface externalization of phosphatidylserine (Annexin-V binding) and preserved mitochondrial transmembrane potential (TMRE) (Figures 4a and c). Blocking of CXCR7 but not CXCR4 decreased the platelet-derived CXCL12-dependent anti-apoptotic effect on monocytes (Figure 4b). Further, neutralizing CXCL12 reduced the anti-apoptotic effect of activated platelet supernatant (APS) (Figure 4b). This indicates that CXCR7 but not CXCR4 appears to be the primary survival receptor for mediating platelet–CXCR12 initiated anti-apoptotic effect on monocytes similar to those observed by recombinant CXCL12 (Supplementary Figure 3).

### Platelets induce differentiation of monocytes into predominantly CD163^+^ macrophages and foam cells through both CXCR4 and CXCR7

**Figure 5:**
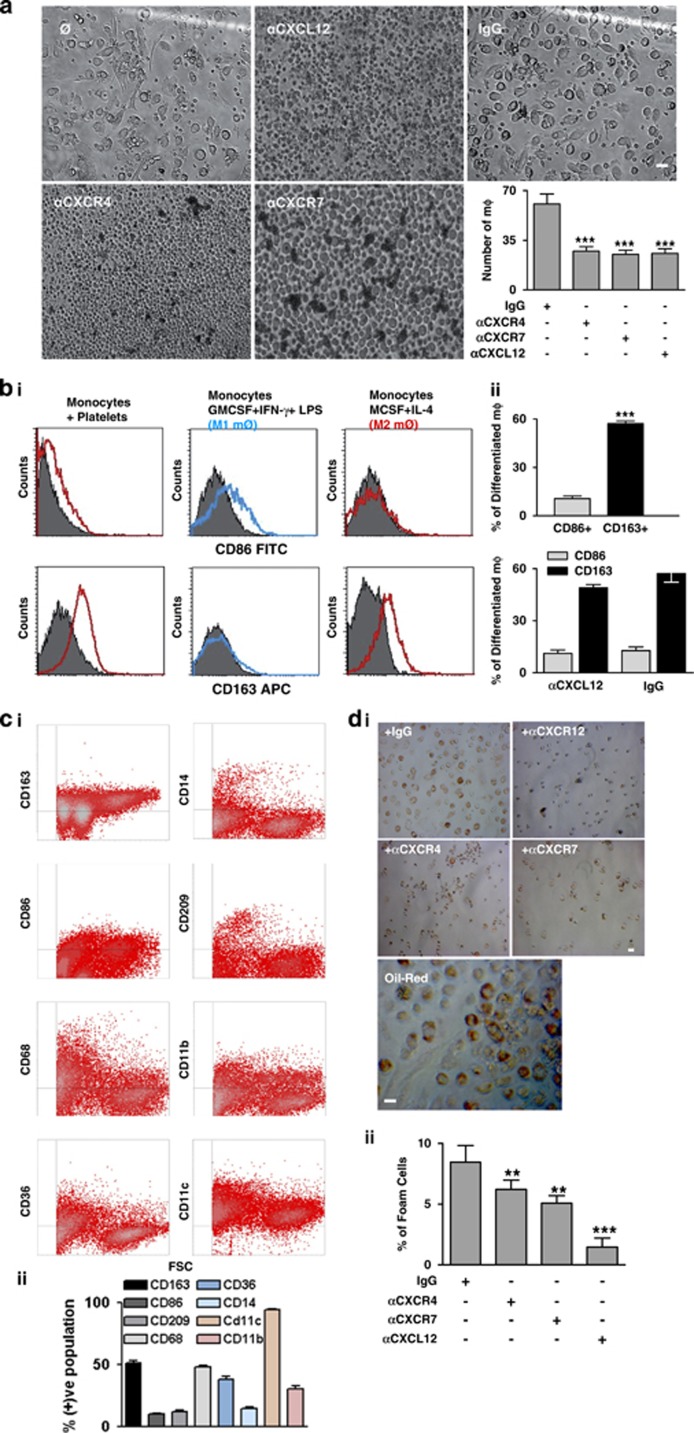
Platelets induce differentiation of monocytes into CD163^+^ macrophages and foam cells through CXCR4–CXCR7. (**a**) Brightfield phase-contrast images from platelet-monocyte co-culture system showing the differentiation of monocytes into macrophages in the presence/absence of neutralizing antibody against platelet-derived-CXCL12 (10 *μ*g/ml) or blocking antibody against CXCR4–CXCR7 (10 *μ*g/ml) with respect to control IgG. Bar=5 *μ*m. **Right**, Bar diagram representing the number of macrophages (mØ) differentiated from a co-culture of platelets–monocytes. The presence of a neutralizing antibody against CXCL12 (***P<0.001 as compared with IgG) in the culture system or blocking antibody against CXCR4–CXCR7 significantly (***P<0.001 as compared with IgG) reduced the number of differentiated macrophages. (**b**) (**i**) Data represent phenotypic characterization of platelet-monocyte co-culture-derived macrophages by flow cytometry. Flow-cytometric histogram overlay for the surface expression of CD86 and CD163 (in red and blue lines as indicated) as overlayed and compared with respective isotype controls (gray fill) showing prominent surface expression of CD163 but not of CD86 in platelet–monocyte co-culture-derived macrophages as compared with culture-derived M2 macrophages (cultured in the presence of M-CSF followed by IL4) which were positive for CD163 but not for CD86 and culture-derived M1 macrophages (cultured in the presence of GM-CSF followed by IFN-*γ* and LPS) which were positive for CD86 but not for CD163. (**ii**) Bar diagram representing phenotypic characterization of platelet–monocyte culture-derived macrophages in terms of CD86, CD163 surface expression which shows a predominantly (***P<0.001 as compared with CD86) CD163 phenotype. **Below**, Bar diagram representing a trend of decrease in the relative % of CD163 macrophages generated in the platelet–monocyte co-culture in presence of a neutralizing antibody against CXCL12. *P*=0.17 as compared with IgG control. Data are mean±S.E.M. from five independent platelet–monocyte co-culture experiments. (**c**) (**i**) Density plots of cells derived from monocyte–platelet co-culture showing the relative abundance of CD163^+^, CD86^+^, CD209^+^, CD68^+^, CD36^+^, CD14^+^, CD11b^+^, CD11c^+^ cells. (**ii**) Bar diagram showing the relative percentage of CD163^+^, CD86^+^, CD209^+^, CD68^+^, CD36^+^, CD14^+^, CD11b^+^, CD11c^+^ cells from platelet– monocyte co-culture. Data are mean±S.E.M. from four independent platelet-monocyte co-culture sets. (**d**) (**i**) Representative images for Oil-red staining of foam cells formed in the co-culture of platelets and monocytes. Bar=5 *μ*m. (**ii**) Bar diagram representing the relative percentage of foam cells generated in platelet–monocyte co-culture, which was significantly (**P<0001 as compared with IgG control) reduced in the presence of blocking antibody against CXCR4–CXCR7 and neutralizing anti-CXCL12 antibody (***P<0001 as compared with IgG control) in culture. Data are mean±S.E.M. from three independent platelet–monocyte co-culture systems

Platelet-derived CXCL12 differentially regulates differentiation of CD34^+^-progenitor cells into a macrophage and foam cell phenotype.^10, 13^ CXCR4–CXCR7 are reported to determine differentiation of macrophages in response to recombinant CXCL12.^24^ Moreover, the relative expression of CXCR4 and CXCR7 also alters during macrophage differentiation in comparison with circulating monocytes as the expression of CXCR4 is reduced and that of CXCR7 is enhanced.^24^ In the present study, we show that co-culture of platelets with monocytes triggered platelet clearance and monocyte differentiation into macrophages (Figure 5). The number of macrophages was significantly reduced in the presence of CXCL12-neutralizing antibody and blocking antibody against CXCR4 and CXCR7 (Figure 5a).

These macrophages were characterized phenotypically by flow cytometry. We found that macrophages generated during platelet/monocyte co-cultures were predominantly CD163^+^ phenotype, and comparable to the culture-derived M2 macrophages (Figure 5bi). Only a small population of cells were positive for CD86 in the platelet–monocyte co-culture (Figure 5b). The relative percentage of other phenotypic markers that were assessed for the mix population of cells derived from monocyte–platelet co-culture is shown in Figures 5b and c. Moreover, the relative percentage of CD163^+^ macrophages was reduced in the presence of CXCL12 neutralization but not in the respective IgG control sets (Figure 5bii). This was comparable to macrophage differentiation from monocyte cultures supplemented with CXCL12 and CXCL11 both of which enhanced the number of macrophages differentiated and in a CXCR4–CXCR7 and CXCR7-dependent manner, respectively (Supplementary Figure 4).

Upon further prolonging the platelet-monocyte co-culture we found that a substantial percentage of macrophages had morphological features of foam cells and were positive for oil-red staining (Figure 5d) indicating the presence of lipid-rich vacuoles. Formation of foam cells was dependent both on platelet-derived CXCL12 and on monocyte CXCR4 and CXCR7 (Figure 5dii). This effect was comparable to monocyte cultures supplemented with Ox-LDL generating foam cells (Supplementary Figure 5). When M1 and M2 pre-differentiated macrophages were co-incubated with platelets for 5 days, the relative percentage of foam cells generated from M2 macrophages was less than from M1 macrophages (Supplementary Figure 6).

### OxLDL induces platelet apoptosis and promotes their phagocytosis by monocyte/macrophages

**Figure 6:**
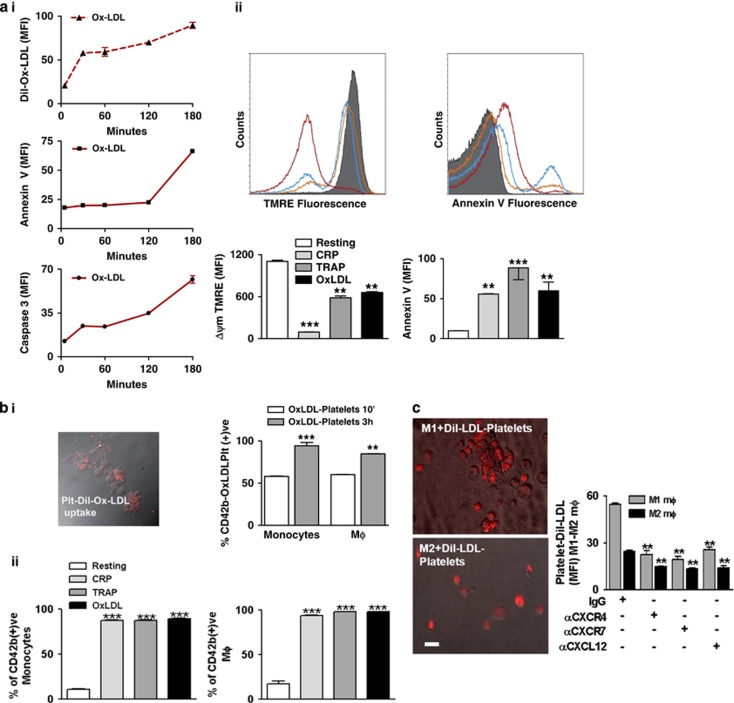
OxLDL-loaded apoptotic platelets are phagocytosed by monocytes and macrophages to form foam cells. (**a**) (**i**) Flow-cytometric data showing uptake of Dil-OxLDL by platelets in a time-dependent manner (0–3 h). **Middle**, Gradual enhancement in surface expression of Annexin V and **Below**, Caspase 3 activity in platelets following uptake of OxLDL in a time-dependent manner (0–3 h). Data are mean±S.E.M. derived from experiments with three healthy platelet donors. (**ii**) Flow-cytometric histogram overlay showing the relative extent of mitochondrial membrane depolarization (Δψm) denoted by TMRE fluorescence and relative extent of annexin V binding among apoptotic platelets treated with OxLDL, TRAP, or CRP. Gray fills indicate fluorescence response from resting platelets, response from OxLDL-treated platelets is indicated by red line, TRAP by blue, and CRP by yellow. **Below**, Bar diagram showing the relative mean fluorescence intensity (MFI) for TMRE and Annexin V-FITC among apoptotic platelets when platelets were activated with OxLDL (20 *μ*g/ml), TRAP (25 *μ*M), or CRP (5 *μ*g/ml). ***P<0.001 and **P<0.01 as compared with resting platelets. Data are mean±S.E.M. from four independent experiments. (**b**) (**i**) Bar diagram showing the relative percentage of monocytes and macrophages following phagocytosis of Ox-LDL-loaded CD42b^+^ platelets after the platelets had been incubated with Ox-LDL for 10 min and 3 h. ***P<0.001 and **P<0.01 as compared with 10 min. Data are mean±S.E.M. from three independent experiments. (**ii**) Bar diagram showing the comparative extent and relative percentage of monocytes and macrophages showing phagocytic uptake of OxLDL-loaded or TRAP- or CRP-treated CD42b^+^ platelets. ***P<0.001 as compared with phagocytic uptake of resting platelets. Data are mean±S.E.M. from four independent experiments. (**c**) Representative immunofluorescence images showing the uptake of Dil-OxLDL-loaded platelets by culture-derived M1 and M2 macrophages when Dil-OxLDL-loaded platelets were introduced into the M1 and M2 culture (Bar=5 *μ*m). Bar diagram from flow cytometric data showing the relative uptake of Dil-OxLDL-loaded platelets by M1 and M2 macrophages which is enhanced upon supplementation with rCXCL12 and reduced in the presence of blocking antibody against CXCR4–CXCR7 and neutralizing antibody for CXCL12. **P<0.01 as compared with respective IgG control. Data are mean±S.E.M. from three independent platelet–monocyte co-culture systems

Platelets accumulate lipids and contribute to macrophages/foam cells development and progression of vascular inflammation and atherogenesis.^25, 26, 27, 28, 29^ OxLDL binds to platelets and induces platelet activation.^25, 26, 27, 28^ Thus, we asked whether uptake of OxLDL by platelets influences their phagocytosis by monocytes/macrophages. We found a substantial time-dependent uptake of OxLDL by platelets (Figure 6a). The uptake was further substantiated upon CXCL12 supplementation and was counter-regulated upon CXCR4–CXCR7 blocking on platelet surface and in the presence of CXCL12 neutralizing antibody (Supplementary Figure 7). Further, OxLDL induced a pro-apoptotic phenotype among platelets in a time-dependent manner as indicated by an increase in phosphatidylserine exposure (‘eat me signal') and caspase-3 activity (Figure 6ai). OxLDL-induced platelet apoptosis was compared with other platelet agonists like TRAP and CRP both of which induced substantial platelet apoptosis as evident from depolarized mitochondrial membrane (TMRE staining) and surface exposure of phosphatidylserine (Annexin V binding) (Figure 6aii). Uptake of OxLDL promoted phagocytosis of these apoptotic platelets by monocytes and macrophages (Figure 6bi). Phagocytic uptake of platelets exposed to OxLDL for a prolonged time was more, corresponding to the level of apoptosis induced in platelets. Phagocytic uptake of OxLDL-treated apoptotic platelets was also compared with TRAP- and CRP-treated apoptotic platelets. Monocytes and macrophages showed comparable phagocytic uptake of all apoptotic platelets irrespective of the apoptotic trigger (OxLDL *versus* TRAP/CRP) as shown in Figure 6bii. Phagocytosis of OxLDL-laden platelets was more in M1 compared with M2-type macrophages and in both subtypes was substantiated by CXCL12, dependent on CXCR4 and CXCR7 (Figure 6c). The platelet uptake potential of M1 macrophages being higher in comparison with M2, the number of foam cells generated was also higher in the co-culture of M1 macrophage-platelets than that of M2 macrophage-platelets (Supplementary Figure 4).

## Discussion

The major findings of the present study are (i) surface expression of CXCR4–CXCR7 is dynamically regulated on migrating monocytes, (ii) CXCR4 but not CXCR7 mediates platelet–CXCL12-dependent monocyte migration, (iii) CXCR7 primarily mediates monocyte adhesion to platelet-derived CXCL12, (iv) CXCL12 promotes phagocytosis of platelets by monocytes and macrophages through both CXCR4 and CXCR7, (v) platelet-derived CXCL12 promotes survival of monocytes against BH3-mimetic-induced apoptosis through CXCR7, (vi) platelets induce differentiation of monocytes primarily into CD163^+^ macrophages through both CXCR4 and CXCR7, and (vii) OxLDL uptake induces platelets apoptosis and facilitates their phagocytic clearance by monocytes, M1–M2 macrophages prompting their subsequent differentiation into lipid-laden foam cells supported by CXCL12–CXCR7. Our findings provide evidence that platelet-derived CXCL12 is an important chemokine through which differential involvement of CXCR4 and CXCR7 regulates monocyte functions, survival, and subsequent differentiation.

Platelets have a regulatory role in inflammation.^1, 2^ Platelets store and release chemokines^1, 2, 3^ such as CXCL12^4, 5, 6, 7^ and interact with a variety of inflammatory cells including monocytes.^29^ Monocytes express CXCR4, which is a cognate receptor for CXCL12. However, the expression of CXCR7 in human or murine monocytes, lymphocytes, and NK cells is widely debated.^19, 24, 30^ Expression of CXCR7 in circulating monocytes or in primary lymphocytes and HSB-2 (lymphoid) cell lines is primarily depicted intracellularly with limited surface expression and characterized by a high inter-individual variability.^19, 24, 30^ Still others demonstrate the relative difference in CXCR7 expression among hematopoietic cells (Mono-Mac, MOLT13, SupT1, and Nalm6) showing low or negative CXCR7 expression and myelo-monocytic cells (HL-60, THP-1, and U937) where CXCR7 is easily detectable.^30^ Currently, we show that monocytes substantially express CXCR4 and CXCR7. Furthermore, surface expression of CXCR4–CXCR7 on monocytes is differentially regulated and dependent on the functional state of monocytes. In response to CXCL12 and MIF but not to MCP-1, surface expression of CXCR7 on migrating monocytes is significantly enhanced as in platelets.^8^ Unlike in platelets which lack the co-receptor CD74, MIF could externalize CXCR7 on CD74 expressing monocytes,^21^ thus emphasizing a cell-specific effect and difference in intracellular signalling mechanism following CXCR4 ligation by MIF. The dynamic trafficking of CXCR4–CXCR7 could also be influenced by the differential release kinetics of CXCL12 and MIF as shown for platelets, with CXCL12 being faster than MIF release, which is detected after prolonged incubation.^31, 32^ However, to date, the role of both chemokine receptors for monocyte function is incompletely understood. We found that CXCL12-dependent migration is primarily regulated by CXCR4 but not by CXCR7.

Contradictory reports exist regarding the involvement of CXCR7 in migration. Previously, involvement of CXCR7 in migration was suggested using a higher concentration of blocking antibody, also with CXCR7 antagonist and siRNA approach in CXCR7-expressing THP-1 cells.^24^ Another study depicting chemotactic responses of THP-1 and U937 cells, also CXCR4^+^CXCR7^+^CXCR3^−^ Nalm6 cells toward CXCL12 and CXCL11 reported a robust response toward CXCL12 but week response toward CXCL11.^30^ Therefore, variability in experimental approach, cell system and basal CXCR7 expression on cells should be considered. Nevertheless, as circulating monocytes have a higher surface expression of CXCR4 than CXCR7 at basal levels, it seems reasonable that the initial chemotactic response is primarily mediated through CXCR4 which drives a dynamic CXCR7 surface enrichment and therefore a more active participation of CXCR7 in the subsequent CXCL12-triggered responses.

Following migration, monocytes interact with CXCL12-enriched inflammatory surfaces such as adherent platelets, which display an adhesion matrix for monocytes. Since activated platelets release substantial CXCL12, interaction of platelet–CXCL12 with monocyte–CXCR4 may be a contributing mechanism for monocyte recruitment at site of platelet adhesion. We found that in contrast to migration, static and dynamic adhesion of monocytes on immobilized CXCL12 surface also over adherent platelets involved CXCR7 engagement and was facilitated by platelet-derived CXCL12. These results document that distinct functions can be assigned to the chemokine receptors (CXCR4 migration, CXCR7 adhesion) in the context of platelet-derived CXCL12. It is tempting to speculate that the initial chemotactic response is primarily mediated through CXCR4, which drives a dynamic CXCR7 surface expression and therefore confers a more proadhesive response to monocytes toward CXCL12-expressing platelets.

CXCL12 and CXCL11 also enhance adhesiveness of myeloid cells to fibronectin-coated surface or to endothelial (HUVEC) layer through CXCR7 involvement.^30^ Activated platelets present an adhesive substrate for monocyte recruitment, which is greatly facilitated by *P*-selectin and monocyte PSGL-1 also by β2-Integrin, CD18, and CD11b.^29, 33, 34, 35, 36^ In the present study, we did not address the role of adhesion receptors in platelet–monocyte adhesion. However, activated platelet-derived CXCL12 appears to be a contributory factor since neutralizing CXCL12 over adherent platelets significantly reduced monocyte adhesion under both static and dynamic flow conditions.

Adherent platelets undergo activation-induced apoptosis and expose the ‘eat me' signal in form of externalized phosphatidylserine on their surface, which is a trigger for their phagocytic uptake by interacting monocytes-macrophages. CXCL12 substantially enhanced phagocytic uptake of platelets by monocytes and M1–M2 macrophages, which was significantly reduced in the presence of CXCR7 blocking on monocytes and particularly M1–M2 macrophages, which have relatively higher expression of CXCR7 than monocytes.^9^ Interestingly, phagocytosis of platelets was dependent on the levels of CXCR7 surface expression, since M1 macrophages (higher CXCR7) revealed a higher platelet phagocytosis activity compared with M2 macrophages (lower CXCR7). However, CXCL12 could elicit a significant enhancement in both subtypes. Previously, we have shown that co-culture of platelets with human CD34^+^ progenitor cells and monocytes results in platelet phagocytosis, causing their differentiation into CD68^+^ macrophages and foam cells.^10, 13^ Currently, we found that monocyte-mediated platelet phagocytosis could largely contribute to the resolution of platelet surface, which was also significantly reduced following CXCL12 neutralization, and blocking of CXCR4–CXCR7 on monocytes.

Phagocytic uptake of platelets counteracts monocyte apoptosis triggered by starvation or CD95, through downregulation of caspase-9 and -3 and upregulation of heat-shock protein 70 during uptake of platelets.^20^ Moreover, the anti-apoptotic effect of platelets is further increased and dependent on their grade of activation, suggesting potential involvement of platelet-derived mediators. CXCL12 is an important survival factor for a variety of cells.^21, 22, 23^ Prolonged survival is an important determinant for differentiation process. Here, we show that platelet-derived CXCL12 significantly counter-regulated ABT-737-induced apoptosis in monocytes upon challenging monocyte with the BH3-mimetic-ABT-737. Activated platelet-derived CXCL12 significantly counter-regulated ABT-737-induced apoptosis in monocytes through active engagement of CXCR7 as previously reported for platelets.^8^ Therefore, following monocyte migration largely mediated through CXCR4, CXCR7 features prominently in mediating adhesion to platelets, phagocytic uptake of platelets and in substantiating monocyte survival.

Previous reports document the heterogeneity of circulating CD14^+^ monocytes, in terms of phenotypic and functional characteristics, also diverse differentiation potential into macrophage-foam cells, dendritic cells, and monocyte-derived multipotential cells (MOMCs).^37^ Currently, we show that platelets are phagocytosed by monocytes in co-culture and platelet-derived CXCL12 through CXCR4–CXCR7 contributed to the differentiation of monocytes predominantly into CD68^+^CD163^+^ macrophages. This is in accordance with previous publication showing CXCL12 promoting differentiation of monocytes in to a VEGF and CCL1 generating pro-angiogenic and immunosuppressive CD68^+^CD163^+^CD209^+^ phenotype.^24^ Macrophage number is substantially reduced following CXCL12 neutralization and blocking the receptors. Since monocytes, THP-1, and U-937 monocytic cells secrete substantial amounts of CXCL12, which exerts an autocrine effect on macrophage differentiation,^24^ the relative contribution of monocyte-derived CXCL12 in the co-culture set up cannot be ruled out.

Activated platelet supernatant induces phosphorylation of Akt and rapid mobilization of intracellular Ca^2+^ via a yet unknown pertussis toxin-sensitive G protein-coupled receptors.^33^ However, the platelet-derived inflammatory mediator had not been defined. Previously, we have shown that platelets store and secrete substantial amounts of CXCL12, which mediates recruitment of circulating CD34+ progenitor cells via G protein-coupled receptor CXCR4.^4, 5^ Binding of CXCL12 to CXCR4 induces Akt phosphorylation in monocytes.^24^ Although we did not address CXCL12-mediated signalling in monocytes in the present study, the herein described stimulation of CXCR4 via platelet-derived CXCL12 might be a mechanism influencing platelet-induced pro-inflammatory response among monocytes.

Platelets influence cell differentiation. Lipid-loaded platelets are phagocytosed by CD34^+^ progenitor cells^13^ or monocytes^38, 39^ and promote formation of foam cells. OxLDL is a major determinant of foam cell generation and platelets bind to OxLDL that activates them.^25, 26, 27, 28^ OxLDL was taken up by platelets in a time-dependent manner, which induced platelet apoptosis like other agonists TRAP and CRP although to a different extent and triggered their phagocytosis by monocytes and macrophages as lipid-rich platelets communicate an ‘*eat me signal*' to the monocyte/macrophages. The phagocytic potential of M1 macrophages was substantially higher compared with M2 and was substantiated by CXCL12 dependent on CXCR4–CXCR7. Thus, platelets represent a major source and vehicle of OxLDL that in turn promotes monocyte/macrophages derived foam cell formation. Ox-LDL itself is an inducer of platelet–monocyte aggregates both *in vitro* and *in vivo*.^25, 26, 27, 28^ Such interaction facilitates OxLDL uptake by monocytes, involving platelet CD36–OxLDL interaction, release of CXCL4 and phagocytosis of platelets.^27, 28, 29^

Activated platelets induce a pro-inflammatory phenotype of monocytes characterized by secretion of cytokines including TNF-*α*, IL8, MCP-1, and IL1b.^29, 34, 35, 36, 40^ Currently, we have shown that the presence of platelets along with culture-derived M1–M2 macrophages promoted their subsequent differentiation into foam cells. Therefore, platelets and platelet-derived-CXCL12 through CXCR4–CXCR7 engagement can drive the differentiation of monocytes into an anti-inflammatory and regenerative M2 phenotype but in the presence of sustained inflammation and M1–M2 macrophages in the immediate microenvironment, platelets can promote their further differentiation into inflammatory foam cells. This mechanism is critical in diseases where vascular inflammation, OxLDL, and foam cell generation is a prominent determinant for disease onset and progression (e.g., atherosclerosis).

In conclusion, our data establish platelet-derived CXCL12 and its receptors CXCR4 and CXCR7 as an important mechanism to regulate monocyte function and macrophage/foam cell differentiation. Targeting this mechanism may be an effective strategy to control inflammation where macrophages/foam cell formation has a prominent role such as vascular inflammation and atherosclerosis.

## Materials and Methods

### Materials

Recombinant murine/human/feline SDF-1*α*/CXCL12, recombinant human MIF, recombinant human MCP-1, mouse monoclonal anti-human CXCR4-PE, mouse anti-human/mouse CXCR7-PE, mouse monoclonal anti human CD86-FITC, mouse monoclonal anti-human CD163-APC, rat anti-mouse CD14-APC, mouse anti-human CD209-PE, mouse monoclonal anti-human CXCR4-unconjugated, mouse monoclonal anti-human CXCR7-unconjugated antibodies were procured from R&D Systems (Abingdon, UK). Anti-human CD11c-APC from Biolegend (San Diego, CA, USA), anti-mouse GPIb*α*-FITC from Emfret (Emfret Analytics, Eibelstadt, Germany), rat anti-mouse Mac-3 unconjugated antibody from BD Pharmingen (San Jose, CA, USA), rat anti-mouse F4/80 unconjugated antibody from AxYll, anti-mouse F4/80-APC from eBiosciences (San Diego, CA, USA), rabbit polyclonal antibody to CXCR7 was from Abcam (Cambridge, UK). Rabbit monoclonal-cleaved active Caspase 3 antibody was purchased from Cell Signaling Technology (Beverly, MA, USA). Alexa Fluor-488 Goat anti rabbit-IgG, Alexa Fluor-647donkey anti-mouse-IgG, Alexa Fluor 555 goat anti-rat IgG, Alexa Fluor 488 donkey anti-rabbit IgG, Alexa Fluor 488 goat anti-rat IgG, and secondary antibodies To-Pro 3 iodide were purchased from Invitrogen (Carlsbad, CA, USA). Anti-human CD42b-FITC, anti-human CD36-FITC, and anti-human CD11b-FITC was procured from Beckman Coulter (Paris, France), and anti-human CD68-FITC was from Dako (Carpinteria, CA, USA). Ficoll was from GE Healthcare (Uppsala, Sweden), TMRE from Invitrogen, FCCP from Abcam Biochemicals (Cambridge, UK) and Annexin V-FITC conjugate was purchased from Immuno Tools (Friesoythe, Germany). ABT-737 was procured from Selleckchem (Houston, TX, USA), Trypan blue from Sigma-Aldrich (St. Louis, MO, USA), Dil-High oxidized human low density lipoprotein (Dil-Ox-LDL) and High oxidized human low density lipoprotein (Ox-LDL) were purchased from Kalen Biomedicals (Köln, Germany), and Horm Collagen was from Takeda Austria GmbH (Linz, Austria). Mouse and rabbit, mouse control IgGs were procured from Santa Cruz Biotechnologies (Dallas, TX, USA) and control rat IgG from Abcam.

### Isolation of peripheral blood monocytes and platelets

Peripheral blood monocytes were isolated using leukocyte buffy coat preparations from healthy donors,^41^ through differential gradient centrifugation in Ficoll gradient (20 min, 800 × *g*), followed by adhesion depletion on plastic surface (0.5 × 10^6^ cells/ml). Non-adherent cells were removed by gentle washing. The remaining adherent cells were harvested. Purity of isolated monocytes was ascertained by FSC-SSC parameters and surface expression of CD14 by flow cytometry (FACS Calibur BD Biosciences, San Jose, CA, USA). Monocytes were cultured in RPMI-1640 supplemented with 10% fetal calf serum, 100 U/ml penicillin, 100 *μ*g/ml streptomycin, and 2 mM L-glutamine at 37 °C and 5% CO_2_ humidified atmosphere.^41, 42^

Washed platelets were isolated from peripheral blood as previously described.^8, 21^ Briefly, blood was collected in acid-citrate-dextrose (ACD)-buffer and centrifuged at 430 × *g* for 20 min. Platelet-rich plasma (PRP) thus obtained was added to Tyrodes-HEPES buffer (HEPES-2.5 mM; NaCl-150 mM; KCl-1 mM; NaHCO_3_-2.5 mM; NaH_2_PO_4_-0.36 mM; glucose- 5.5 mM; BSA-1 mg/ml; pH 6.5) and centrifuged at 900 × *g* for 10 min. The platelet pellet was suspended in Tyrodes-HEPES buffer (pH 7.4; supplemented with CaCl_2_-1 mM; MgCl_2_-1 mM). Activated platelet supernatant enriched in CXCL12 was generated from activated platelets adherent on collagen (100 *μ*g/ml) coated surface for 1 h at room temperature (RT). Thereafter, platelets were centrifuged at 900 × *g* for 5 min and activated platelet supernatant enriched in platelet-derived CXCL12 was collected. Generation of platelet supernatant from non-activated (resting) platelets followed the same procedure in the absence of collagen-coated surface or any other platelet agonist.

### Expression of CXCR4–CXCR7 in monocytes

Western blot analysis to detect the expression of CXCR4 and CXCR7 in monocytes was performed as reported previously^8^ using untreated resting monocytes isolated from three healthy donors.

### Differential trafficking of CXCR4 and CXCR7 in monocytes

#### Flow cytometry

Monocytes were treated with rCXCL12 (1 *μ*g/ml) at RT for 20 min and stained for surface expression of CXCR4–CXCR7 with respective fluorochrome-conjugated antibodies and analyzed by flow cytometry.^8, 21^

#### Immunofluorescence confocal microscopy

Monocytes were kept untreated or pre-treated with rCXCL12 (1 *μ*g/ml) for 20 min at RT. Thereafter, samples were fixed with 2% paraformaldehyde, applied to 0.01% poly-L-lysine coated coverslips and permeabilized with 0.3% Triton X-100. Following blocking with 1% BSA-PBS for 1 h at RT, samples were labelled overnight at 4 °C with respective primary antibodies-mouse anti-human CXCR4 (1 : 50) and rabbit anti-human/mouse CXCR7 (1 : 50). After washing with washing buffer (PBS+0.3%Triton X-100+0.1% Tween-20), samples were incubated with the corresponding secondary antibodies (Alexa Fluor 647 donkey anti-mouse IgG at 1 : 200, Alexa Fluor 488 goat anti-rabbit IgG at 1 : 100) for 2 h at RT, washed and the coverslips were mounted with antifade fluorescence mounting medium (Dako). Images were acquired using a Zeiss LSM 510 Meta, Axioplan 2 Imaging Confocal Laser Scanning Microscope (Zeiss, Oberkochen, Germany) with a × 100 ocular.^8, 21^

### Monocyte chemotaxis

Migration of monocytes toward platelet-derived CXCL12, CXCL12, MIF, and MCP-1 was analyzed in a modified Boyden chamber (Neuro Probe Inc., Gaithersburg, MD, USA) with a 5-*μ*m pore polycarbonate membrane separating the upper from the lower compartment. Monocytes (2 × 10^4^cells/well in RPMI-1640 medium) were loaded onto the upper chamber, and resting (RPS) or activated (APS) platelet supernatant (diluted 1 : 1 in RPMI-1640) or recombinant proteins served as chemo-attractants in the lower chamber. Neutralizing antibody against platelet-derived-CXCL12 or respective isotype control (10 *μ*g/ml) was also added along with APS/RPS as indicated. CXCL12 receptors CXCR4 and CXCR7 were blocked on monocytes for 30 min at RT with mouse anti-human CXCR4 (10 *μ*g/ml) or mouse-anti-human CXCR7 (10 *μ*g/ml) and respective IgG controls before chemotaxis. After incubation for 6 h at 37 °C and 5% CO_2_, humidified atmosphere, the membrane was fixed with 100% ethanol and stained with May-Grünwald/Giemsa. The membrane was mounted on glass slides; the number of migrated cells was counted for each well in randomly selected microscopic fields at × 20 magnification.^41^ Relative surface expression of CXCR4 and CXCR7 among the migrated monocytes in the lower chamber was analyzed by flow cytometry.

### Monocyte chemotaxis toward collagen adherent platelets in transwell migration chamber

Washed platelets adhered to collagen (100 *μ*g/ml coated for overnight at 4 °C) coated surface for 1 h at RT in the lower chamber of a transwell migration setup. Monocytes isolated from healthy donors were labelled with 5-(and-6)-carboxyfluorescein diacetate, succinimidyl ester (5(6)-CFDA) in the dark for 10 min, washed and either kept untreated for treated with blocking antibodies against CXCR4 or CXCR7 or respective IgG controls for a further 30 min. Collagen adherent activated platelet-derived CXCL12 was neutralized where specified with a CXCL12 neutralizing antibody (10 *μ*g/ml of respective IgG control for 30 min) before starting migration. Fluorescently labelled monocytes were loaded on to the upper chamber and migration was carried out for 6 h at 37 °C. At the end of incubation period, the transmigrated monocytes in suspension medium in the lower chambers were collected and their number evaluated by flow cytometry. The lower chamber with collagen adherent platelets and monocytes adhering to activated platelet surface was washed with medium to get rid of the non-adherent cells. After subsequent washing the fluorescence signal from monocytes adherent to platelet surface was estimated by reading the plate with a GloMax Multi-detection System (Promega, Madison, WI, USA) fluorescence plate reader.

### Monocyte adhesion

Monocytes (2 × 10^4^/ml) were kept untreated or pre-treated with blocking antibody against CXCR4 or CXCR7 for 30 min before introducing them to CXCL12-(200 ng/ml)-coated surface. CXCL12 was neutralized where specified with neutralizing antibody against CXCL12 before adding monocytes. In another set of experiment, monocytes were pre-labelled with 5(6)-CFDA for 10 min in dark, washed once before introducing them to rCXCL12-coated surface. After 2-h incubation non-adherent monocytes were washed off and adherent monocytes were quantified by brightfield microscopy. Images were captured from four different randomly chosen optical fields for each sample in a 24-well culture plate (each experimental set having four samples in one plate for every donor) with Zeiss Axio-Vision software (Zeiss). Number of adherent monocytes was counted using ImageJ software (National Institutes of Health, New York, NY, USA). For labelled monocytes, the fluorescence intensity of monocytes adhering to rCXCL12-coated surface was estimated with the GloMax Multi-detection System (Promega) fluorescence plate reader. Fluorescent images of adherent monocytes to CXCL12-coated surface were acquired with Zeiss Aviovert 200 microscope (Oberkochen, Germany) using a × 20 objective with Axiovision software.

### Monocyte adhesion to activated platelets

Washed platelets were allowed to adhere to collagen- (100 *μ*g/ml) coated surface for 1 h at RT. Monocytes (2 × 10^4^/ml) were pre-treated for 30 min with blocking antibody against CXCR4/CXCR7 (or respective IgG control) and added to adherent platelet surface. Platelet-derived CXCL12 was neutralized where specified with neutralizing antibody against CXCL12 (or respective IgG control). For static adhesion after 2 h incubation at 37 °C, non-adherent monocytes were washed off with PBS and number of monocytes adherent to platelet surface was evaluated as stated above.^41^ In another set of experiment, monocytes were pre-labelled with 5(6)-CFDA for 10 min in dark, washed once before introducing them to collagen adherent platelet surface. Static adhesion was carried out for 2 h at 37 °C, following which non-adherent monocytes were washed off and the fluorescence intensity of monocytes adhering to collagen adherent platelets was estimated with the GloMax Multi-detection System (Promega) fluorescence plate reader. Fluorescent images of monocytes adherent to activated platelets were acquired with Zeiss Aviovert 200 microscope using a × 20 objective with Axiovision software. For dynamic adhesion experiments, monocytes (2 × 10^5^/ml) were perfused over adherent platelet surface at arterial shear rates (2000/s). All experiments were recorded in real time on video-CD and evaluated off-line. Number of adherent monocytes was determined as described.^41^

### Culture of M1–M2 macrophages

Macrophages were differentiated from peripheral blood monocytes. M1 macrophages were generated by culturing monocytes for 7 days in RPMI-1640 supplemented with 20 ng/ml GM-CSF, and stimulating for 24 h with 20 ng/ml IFN-*γ* and 1 ng/ml LPS. M2 macrophages were obtained by culturing with 20 ng/ml M-CSF for 7 days followed by 20 ng/ml IL-4 for 48 h.^40^ Phenotypic characteristics of culture-derived macrophages were assessed by flow cytometry using fluorescently conjugated antibodies against CD86, CD163, CXCR4, and CXCR7.^40^

### Phagocytic uptake of platelets by monocytes and M1–M2 macrophages

#### Flow cytometry

Washed platelets labelled with platelet-specific marker CD42b-FITC were added to monocytes, M1 or M2 macrophages at a ratio of 1 : 200 in RPMI-1640/FCS 10% in the presence of CXCL12, neutralizing antibody against CXCL12 or CXCR4/CXCR7 blocking antibodies (or respective IgG control) and incubated for 4 h at 37 °C and 5% CO_2_ humidified atmosphere. Samples were fixed in 1% paraformaldehyde and analyzed by flow cytometry. Trypan blue (10%) was used to quench for surface fluorescence coming from adherent (but not phagocytosed) platelets and analyzed for CD42b^+^ monocyte/macrophage population, which have phagocytosed platelets and therefore give only intracellular signal. Phagocytic uptake of platelets was evaluated as % of CD42b^+^ monocytes/macrophages.^20^

#### Immunofluorescence confocal microscopy

For immunofluorescence analysis, at the end of 4-h incubation, cells were fixed with 2% paraformaldehyde and applied to 0.01% poly-L-lysine coated coverslips. Nuclear staining for the monocytes was performed with TO-PRO-3 iodide for 30 min in the dark. Thereafter, samples were washed and the coverslips were mounted with antifade fluorescence mounting medium, and confocal microscopic analysis was performed as described above.^8, 20, 21^

#### Dynamic imaging of platelet phagocytosis by monocytes

Platelets were labelled with fluorochrome celltracker orange CMTMR (15 *μ*M) and co-incubated with monocytes. Uptake of red-labelled platelets by monocytes was analyzed over time (2, 4, 8, and 16 h) as described.^13^**

#### Phagocytic clearance of thrombus area

Whole blood from healthy human subjects was perfused through a transparent flow chamber (slit depth 50 *μ*m) over a collagen-coated surface (100 *μ*g/ml) at arterial (1700/s) wall shear rates for 5 min as described previously to generate sufficient thrombus surface coverage.^21^ Monocytes (5 × 10^4^/ml) were incubated on thrombus at 37 °C for overnight. Following incubation period, pictures were taken from 4 to 5 different microscopic areas (Carl Zeiss, optical objective × 20). Analysis was done with AxioVision software (Carl Zeiss, Axiovert 200) and the thrombus area was determined^21^ and data are represented as a bar diagram.

### Monocyte apoptosis

#### Flow cytometry

Monocytes were triggered to undergo apoptosis in response to BH3-mimetic-ABT-737 (25 *μ*M) for 2 h at 37 °C in the presence or absence of supernatant obtained form resting (RPS) and activated (APS) platelets. Monocytes were pre-incubated for 1 h with APS/RPS before apoptosis induction in the presence/absence of neutralizing anti-CXCL12 antibody. As indicated monocytes were pre-treated with anti-CXCR4, anti-CXCR7, or IgG control mAb. Apoptosis was defined with flow cytometry by staining with TMRE denoting mitochondrial transmembrane potential loss (Δψm) and Annexin V binding indicating externalization of phosphatidylserine.^43^

#### Caspase-3 activation

Immunofluorescence labelling was performed to ascertain the presence of cleaved active Caspase 3 in monocytes undergoing apoptosis. Apoptotic monocytes (25 *μ*M ABT-737 induced) treated in the presence/absence of RPS/APS/rCXCL12 or blocking antibody against CXCR4–CXCR7 were fixed with 2% paraformaldehyde, applied to poly-L-lysine-coated coverslips, permeabilized and subsequently stained with the primary antibody against cleaved active Caspase 3 (1 : 100) and counter-stained with Alexa Fluor 488-tagged goat anti-rabbit IgG (1 : 100). Thereafter, coverslips were mounted with antifade fluorescence mounting medium and analyzed by confocal laser scanning microscopy.^43^

### Differentiation into macrophages and foam cells

#### Co-culture conditions

Monocytes and platelets were co-cultured in RPMI-1640 medium supplemented with 10% fetal calf serum, 100 U/ml penicillin, 100 *μ*g/ml streptomycin, and 1 mM L-glutamine at 37 °C and 5% CO_2_ humidified atmosphere for 10 days (for macrophage differentiation) or 15 days (for foam cell) as described.^13, 42^ In some experiments, culture-derived M1/M2-macrophages were co-cultured with platelets for 5 days in the presence of neutralizing antibody against CXCL12, CXCR4, CXCR7, and their respective isotype IgG controls. Differentiated macrophages/foam cells (stained with Oil-red for foam cells) were visualized under the microscope. Images of each experimental culture set-ups were captured from four different optical fields and the number of differentiated macrophages counted during post acquisition image analysis using ImageJ software (National Institutes of Health). Phenotypic characterization of macrophages was done following harvest of cells by flow cytometry using fluorescent-conjugated antibodies against CD14, CD68, CD36, CD11b, CD11c, CD209, CD86 and CD163 with respect to isotype controls. The mix population of cells derived from monocyte–platelet co-culture were gated using FSC-SSC characteristics and the relative percentage positivity for each phenotypic marker was evaluated against their respective isotype control in a density plot quadrant analysis.

### Ox-LDL uptake and phagocytosis of OxLDL-loaded platelets

Platelets were loaded with Dil(1,1′-Dioctadecyl-1-3,3,3′,3′-tetramethyl-indocarbocyanin-perchlorate)-OxLDL (10 *μ*g/ml) for 0–3 h and analyzed by flow cytometry for Dil-OxLDL uptake. Dil-OxLDL-induced apoptosis in platelets was evaluated by externalization of phosphatidylserine (Annexin V binding) as the ‘eat me' signal and Caspase 3 activity as reported previously.^8, 21^ Induction of platelet apoptosis by OxLDL was compared with other platelet agonist like TRAP (25 *μ*M) and collagen-related peptide (CRP-5 *μ*g/ml) by incubating platelets simultaneously with OxLDL (20 *μ*g/ml), TRAP and CRP and then analyzed for apoptosis marker Annexin V binding and mitochondrial transmembrane potential loss (Δψm) using TMRE staining by flow cytometry.

Ox-LDL-loaded platelets were incubated with monocytes, M1 or M2 macrophages for 4 h at 37 °C and analyzed by flow cytometry, quenching for surface fluorescence with Trypan blue. For flow-cytometric analysis, the cells were harvested and looked for CD42b^+^ and Dil-Ox-LDL^+^ macrophages and monocytes in the presence of trypan blue (quenching for surface bound but not phagocytosed platelets). Dil-OxLDL accumulation and phagocytosis of lipid-laden platelets by monocytes and macrophages was also visualized by fluorescence microscopy. Phagocytic uptake of OxLDL-treated apoptotic platelets subsequently labelled with CD42b-FITC was compared with the phagocytic uptake of TRAP- and CRP-treated platelets by monocytes and macrophages following an incubation of 4 h at 37 °C and analyzed by flow cytometry, quenching for surface adherent (but not yet phagocytosed) platelets with trypan blue as stated before.

### Peritonitis model in mice

In all, 10- to 12-week-old C57Bl/6J mice (Charles River, Wilmington, MA, USA) were injected i.p. with 500 *μ*l 4% Thioglycollate (Sigma-Aldrich) to induce peritotinitis or NaCl as a control. The mice were killed 24 h after and the peritoneal lavage was collected by washing the peritoneal cavity with PBS (Sigma-Aldrich) to harvest the infiltrated or migrated cells in response to peritoneal infection.^44^ All animal experiments were conducted according to German law for the welfare of animals and were approved by local authorities.

### Detection of infiltrating cells and CXCL12–CXCR4–CXCR7 surface expression by flow cytometry

The peritoneal lavage collected was centrifuged to harvest the infiltrated or migrated cells. The number of cells was counted. In all, 0.2 × 10^6^ cells were used for each sample. Migrated cells were labelled with respective fluorochrome-conjugated antibodies rat anti-mouse-CD42b-FITC for platelets, anti-mouse-CD14-APC, rat anti-mouseF4/80-APC for monocytes–macrophages, anti-CXCL12-FITC, anti-mouse CXCR4-FITC, and anti-mouse/human CXCR7-PE for 30 min in the dark and fixed in 1% paraformaldehyde and analyzed by flow cytometry. Similarly, whole blood cells were labelled and fixed to be analyzed by flow cytometry. Lavage cells were gated using FSC-SSC characteristics, peripheral blood monocytes were gated for CD14^+^ population and relative percentage of each population, also the percentage of monocyte platelet aggregates, the relative surface expression of CXCL12, CXCR4, and CXCR7 was determined.

### Immunofluorescence confocal microscopic analysis of peritoneal lavage cells

Peritoneal lavage cells were fixed with 2% paraformaldehyde, applied to 0.01% poly-L-lysine coated coverslips and permeabilized with 0.3% Triton X-100. Following blocking with 1% BSA-PBS for 1 h at RT, samples were labelled rat anti-mouse GPIb (1 : 50), rat anti-mouse Mac-3 (1 : 10), rat anti-mouse F4/80 (1 : 100), rabbit anti mouse/human-CXCL12 (1 : 100), rat-anti-mouse CXCR4 (1 : 25), and rabbit anti mouse/human-CXCR7 (1 : 100), and incubated overnight at 4 °C in a humidified chamber. After washing with washing buffer (PBS+0.3%Triton X-100+0.1% Tween-20), samples were incubated with the corresponding secondary antibodies (Alexa Fluor 555 goat anti-rat IgG at 1 : 200, Alexa Fluor 488 donkey anti-rabbit IgG at 1 : 100 and Alexa Fluor 488 goat anti-rat IgG at 1 : 100) for 2 h at RT. After thorough washings, the samples were stained with TO-Pro3-Iodide for nuclear staining for 30 min in the dark, washed thoroughly and mounted with fluorescence mounting medium (Dako). Immunofluorescence images were captured using Zeiss LSM 510 Meta, Axioplan 2 Imaging Confocal Laser Scanning Microscope with a × 100 ocular.^19^

### Statistical analysis

Data are presented as mean±S.E.M. All data were tested for significance using GraphPad Prism software (GraphPad Software, Inc., La Jolla, CA, USA) setting statistical significance at *P*<0.05 with one-way ANOVA using Tukey *post hoc* test while comparing multiple data sets and using unpaired *T*-test while comparing two sets of data.

## Abbreviations

SDF-1: stromal cell-derived factor-1
CXCL12-: chemokine (C-X-C motif) ligand 12
CXCL11: chemokine (C-X-C motif) ligand 11
CXCR4: CXC chemokine receptor 4
CXCR7: CXC-chemokine receptor 7
MCP-1: monocyte chemoattractant protein-1
MIF: macrophage migration inhibitory factor
TRAP: thrombin receptor activating peptide
CRP: collagen-related peptide
APS: activated platelet supernatant
RPS: resting platelet supernatant
IgG: immunoglobulin G
TMRE: tetramethylrhodamine ethyl ester
OxLDL: oxidized LDL
HUVEC: human umbilical vein endothelial cells
PSGL-1: P-selectin glycoprotein ligand
GM-CSF: granulocyte macrophage colony stimulating factor
M-CSF: macrophage colony stimulating factor
IL-4: interleukin 4
IFN-*ϒ*: interferron gamma
GPIb: glycoprotein Ib
