# Supplementary figures and images for: Platelet-derived CXCL12 regulates monocyte function, survival, differentiation into macrophages and foam cells through differential involvement of CXCR4–CXCR7
Source: Cell Death Dis. 2015 Nov 19;6(11):e1989–. doi: 10.1038/cddis.2015.233 (PMC4670914; doi:10.1038/cddis.2015.233)

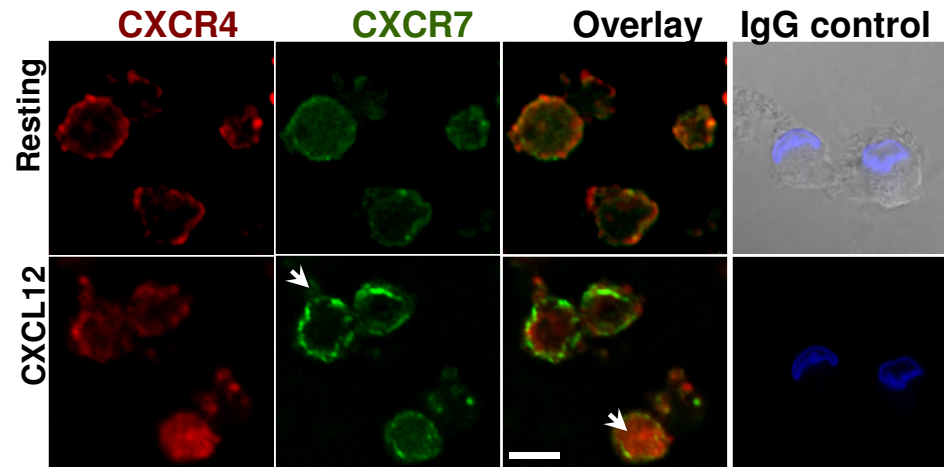

Supplemental Figure 1

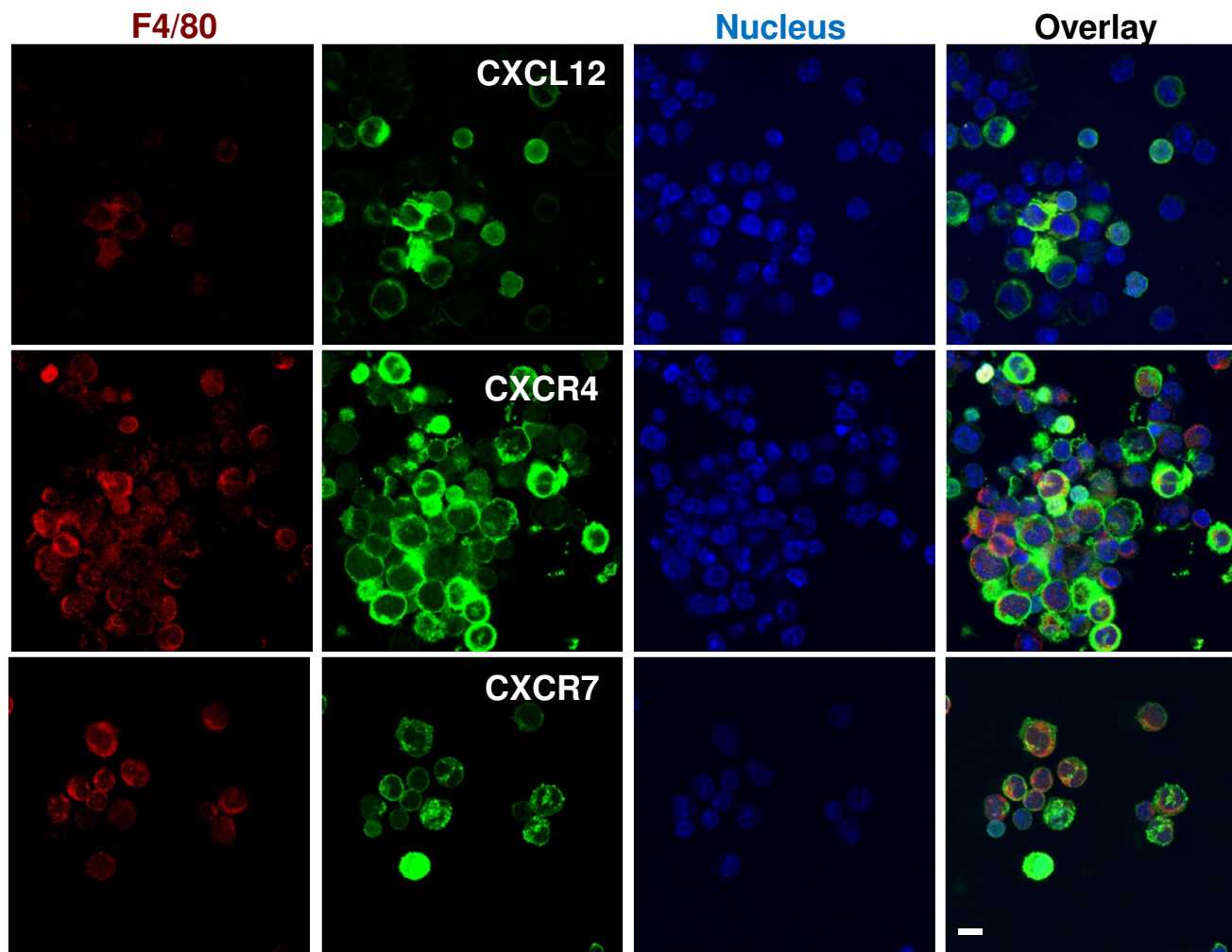

Supplemental Figure 2

Supplement: Supplementary Figures [file cddis2015233x1.pdf]
