# Supplementary material for: Platelet-derived CXCL12 regulates monocyte function, survival, differentiation into macrophages and foam cells through differential involvement of CXCR4–CXCR7
Source: Cell Death Dis. 2015 Nov 19;6(11):e1989–. doi: 10.1038/cddis.2015.233 (PMC4670914; doi:10.1038/cddis.2015.233)
Supplement: Supplementary Figures [file cddis2015233x2.pdf]

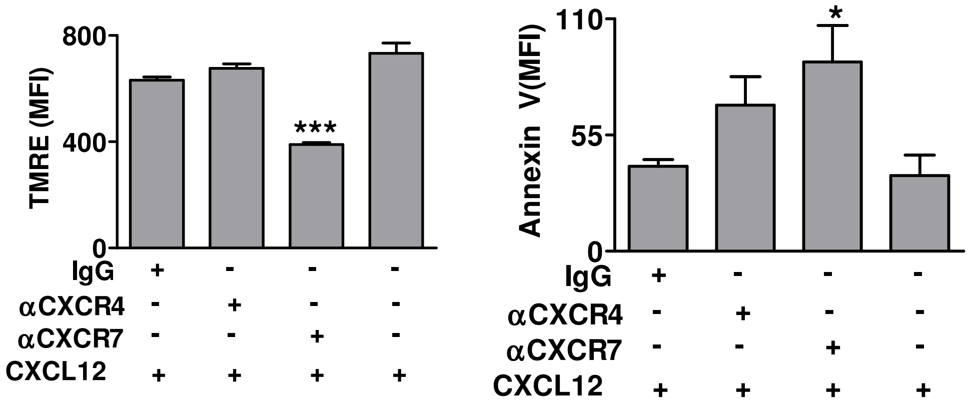

Supplemental Figure 3

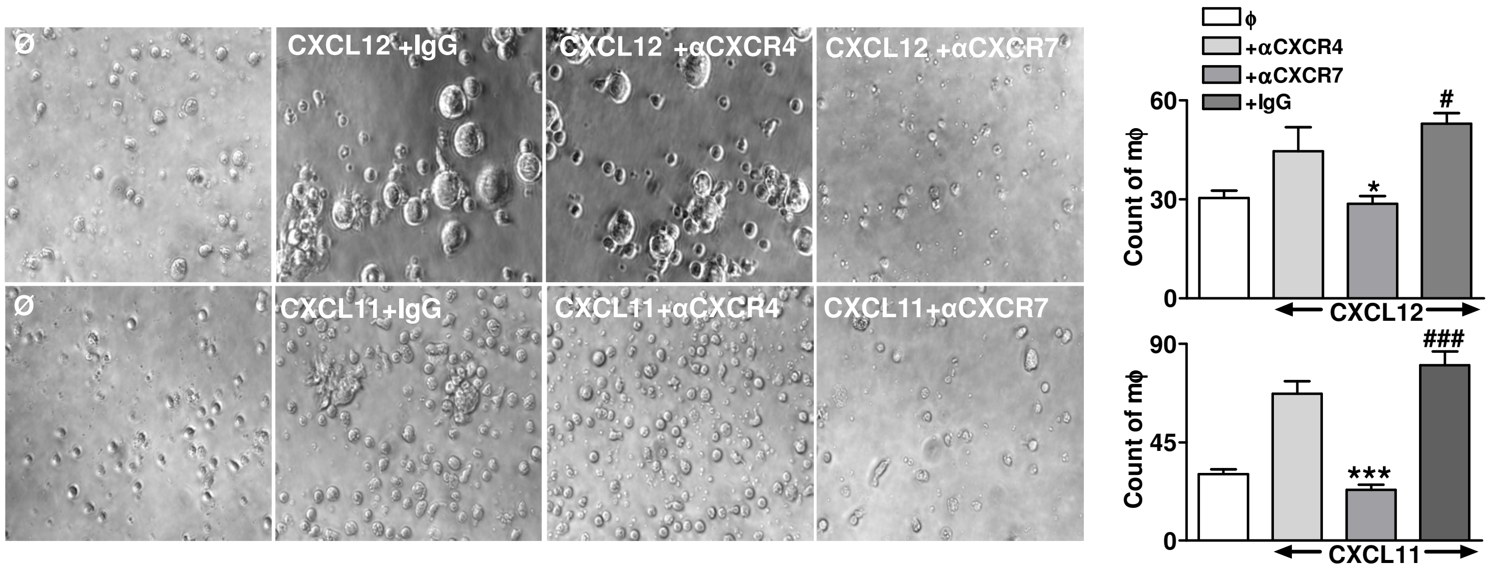

Supplemental Figure 4

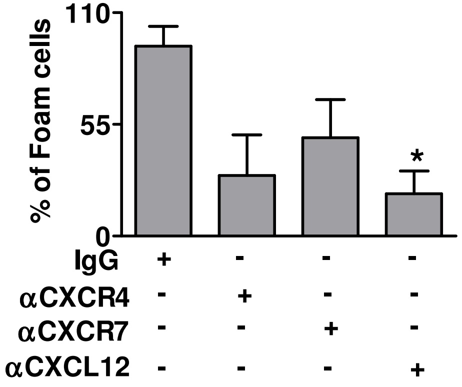

Supplemental Figure 5

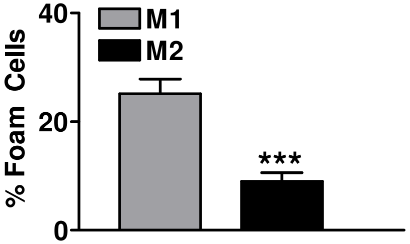

Supplemental Figure 6

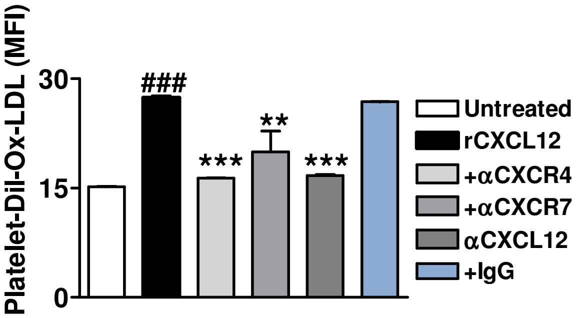

Supplemental Figure 7
